# Remote STrOke Rehabilitation (ReSTORe): study protocol for a randomised controlled trial

**DOI:** 10.1186/s13063-026-09952-5

**Published:** 2026-08-13

**Authors:** Stuart Ennis, Lucy Eggleston, Nicole De Valliere, Harbinder Sandhu, Julie Bruce, Ranjit Lall, Anower Hossain, Mariam Ratna, Jason Madan, Mandana Zanganeh, Yufei Jiang, Jo Parsons, Yin-Ling Lin, Frances Ford, Marcus Davies, Rebecca Palmer, Ali Ali, Gordon McGregor

**Affiliations:** 1 Warwick Clinical Trials Unit, Warwick Medical School, University of Warwick, Coventry, UK; 2 Birmingham Centre for Evidence and Implementation Science, University of Birmingham, Birmingham, UK; 3 Patient and Public Involvement Representative, Sheffield, UK; 4 School of Medicine and Population Health, University of Sheffield, Sheffield, UK; 5 Sheffield Teaching Hospitals NHS Foundation Trust, Sheffield, UK; 6 Institute of Cardio-Metabolic Medicine, University Hospitals Coventry & Warwickshire NHS Trust, Coventry, UK

**Keywords:** Physical health, Mental health, Quality of life, Fatigue, Fitness, Online, Exercise, Psychological support

## Abstract

**Background:**

At least one third of long-term stroke survivors living at home experience ongoing (≥6 months) mild to moderate disability including fatigue, low functional capacity, muscle weakness, anxiety, and depression. Health-related quality of life (HRQoL) and societal participation are affected, particularly after routine contact with health professionals ends. Outpatient centre-based exercise rehabilitation programmes can improve physical and mental health, but these services are rarely provided in the UK. As an alternative, online group physical and mental health rehabilitation is feasible and acceptable to people with long-term conditions and may support recovery for people with stroke. This trial aims to assess the clinical and cost-effectiveness of a supervised, live online, group exercise-based rehabilitation programme (ReSTORe) to support long-term physical and mental health recovery versus best-practice usual care, for people with mild to moderate disability, 6–36 months post-hospital discharge after stroke.

**Methods:**

ReSTORe is a pragmatic, decentralised, two-arm RCT with internal pilot and embedded economic evaluation and qualitative study. Six hundred adults with long-term mild to moderate physical and/or mental health disability after stroke (exclusions: exercise contra-indicated, severe physical disability, mental health/cognitive impairment, not living at ‘home’) will be randomised (1:08:1) to the ReSTORe intervention or control. The ReSTORe intervention consists of (1) 1:1 individual assessment online; (2) 10-week, once-weekly, remote group exercise programme supervised live online; (3) six online group psychosocial and motivational support sessions. The control intervention is best-practice usual care with a single 30-min 1:1 practitioner appointment and general advice on physical activity. The primary outcome is HRQoL with the Patient-Reported Outcomes Measurement Information System (PROMIS) 29+2 Profile v2.1 Preference score (PROPr) at 6 months post-randomisation. Secondary outcomes include PROMIS sub-scores: depression, fatigue, sleep, pain, physical function, social role; health utility; recurrent stroke; and health and social care use. A purposive sample of participants (*n* ~ 30–50) and practitioners (*n* ~ 8) will be interviewed to explore experiences of the trial, outcomes, and interventions.

**Discussion:**

ReSTORe is the largest trial to date testing online exercise-based rehabilitation after stroke. The decentralised trial design will support inclusivity and diversity by helping to ensure productive engagement with underrepresented populations.

**Trial registration:**

ISRCTN (ISRCTN81848233). Registered on 23 January 2025.

**Supplementary Information:**

The online version contains supplementary material available at https://doi.org/10.1186/s13063-026-09952-5.

## Introduction

### Background and rationale {9a}

At least one third of long-term stroke survivors living at home experience ongoing (≥6 months) mild to moderate disability including debilitating fatigue, low functional capacity, muscle weakness, anxiety, and depression [1]. In the UK, this equates to approximately 500,000 people, about a quarter to a third of which are of working age [2]. Health-related quality of life (HRQoL) and societal participation are profoundly affected [3]. After the period of scheduled contact with National Health Service (NHS) health professionals ends (≥6 months), effective treatments for physical and mental health are lacking. In long-term stroke survivors with mild to moderate physical and/or mental health disability, there is considerable morbidity that remains unaddressed following cessation of conventional stroke rehabilitation and care pathways [1]. The number of long-term survivors is increasing due to an ageing population and because treatments have led to better survival rates [4]. It is projected that the annual number of strokes in the UK will increase by 60% between 2015 and 2035 and the number of stroke survivors will double [4].

After stroke, exercise-based rehabilitation programmes, delivered in outpatient centres soon after hospital discharge, can result in short-term improvements in fatigue, physical and mental health, and HRQoL [5], but these services are rarely provided in the UK. Programmes typically involve both resistance training and cardiorespiratory exercise, supervised by exercise specialists, and can range in duration from 6 weeks to 6 months, commonly two to three times per week. A 2021 systematic review [5] of exercise-based rehabilitation after stroke (*n* = 1836; 30 studies) found a small to moderate benefit in HRQoL over 3 to 6 months (standardised mean difference, SMD −0.23 [95% confidence interval, CI, −0.40 to −0.07]) which diminished in the longer term (>9 months) (SMD −0.11 [95% CI, −0.26 to 0.04]). In the short term, physical (SMD −0.33 [95% CI, −0.61 to −0.04]) and mental health (SMD −0.29 [95% CI, −0.49 to −0.09]) improved. Dedicated psychosocial and behavioural interventions were rarely delivered alongside exercise rehabilitation despite some evidence of benefit [6].

As a low-resource, high-capacity alternative to centre-based exercise rehabilitation programmes, online group physical and mental health rehabilitation has proven feasible and acceptable to people with long-term conditions [7, 8]. In the REGAIN trial (*n* = 585), supervised, live online, home-based, group rehabilitation was cost-effective [9] and clinically effective [10] compared to usual care for improving HRQoL (PROMIS-PROPr; 0.03 [0.01 to 0.05] *p* = 0.015) and fatigue (PROMIS fatigue sub-scale; 2.5 [1.19 to 3.81] *p* < 0.001) in people with long-term physical and mental health sequelae after COVID-19 hospital discharge. Supervised home-based exercise rehabilitation programmes can also improve fatigue and HRQoL in other long-term conditions, e.g. cancer and multiple sclerosis [11, 12].

People with severe disability after stroke are commonly supported by long-term holistic health and social care systems [1]. The long-term physical and mental health needs of those with mild to moderate disability, however, may not be sufficiently managed, most likely due to the inability of the healthcare system to support demand. A clinically and cost-effective supervised online exercise-based rehabilitation programme has the low-resource infrastructure and capacity to reach and support large numbers of long-term stroke survivors in the UK, contributing to their ongoing recovery and effective return to societal participation and economic productivity.

Exercise-based rehabilitation delivered online can improve fatigue and HRQoL in clinical populations, but there are no data for long-term stroke survivors. A definitive trial testing the clinical and cost-effectiveness of a remote online exercise-based rehabilitation intervention—‘Remote STrOke Rehabilitation’ (ReSTORe)—for long-term stroke survivors with mild to moderate physical and/or mental health disability is needed.

### Explanation for the choice of comparator {9b}

We recognise the challenges of recruiting people to studies where the usual care arm receives no additional treatment or care; our patient partners consistently raise this as an issue of concern and inequity. Therefore, for our control arm, we will deliver a ‘*best-practice usual care*’ intervention. This will comprise an individual ReSTORe specialist appointment, with general advice on safe physical activity and living with stroke, supplemented with freely available Stroke Association leaflets, including aphasia-friendly and translated versions. A 30-min 1:1 online appointment will allow the practitioner to discuss individualised ways in which the participant can independently engage in self-directed physical activity. Participants will not be provided with a structured exercise plan; rather comprehensive information detailing ways in which physical activity can be incorporated into their everyday lives. No specific psychological techniques will be used when providing this information. This allows us to offer the control group best-practice usual care, whilst retaining the aim of the study comparing a group who receive a supervised online rehabilitation programme with a group who do not.

### Objectives {10}

The aim of the trial is to assess the clinical and cost-effectiveness of a supervised, live online, group exercise-based rehabilitation programme (ReSTORe) to support long-term physical and mental health recovery versus best-practice usual care, for people with mild to moderate disability, 6–36 months post-hospital discharge after stroke.

The objective is to run a definitive RCT testing the clinical and cost-effectiveness of the ReSTORe intervention versus a single session of advice including:

1. Pre-pilot to adapt, refine, and test complex intervention materials, delivery, and practitioner training, and prepare trial set-up.
2. Internal pilot with formative process evaluation to test recruitment and trial procedures.
3. Main trial with embedded process evaluation.

## Methods: patient and public involvement, and trial design

### Patient and public involvement {11}

The ReSTORe intervention and trial procedures were developed during the grant funding application process and subsequently during the pre-pilot phase. Patient and public involvement was pivotal at every stage. Lay partners and co-applicants were fully integrated into every stage of trial development, taking an active role in refining intervention components and reviewing the application. Two lay co-applicants sit on the monthly TMG meetings and have a key role in steering the conduct of the trial. They reviewed trial documentation, e.g. participant information leaflet, to ensure user appropriateness, in particular accessibility for those with communication impairments. Lay partners have been actively involved in trial publicity and will help with the dissemination of findings through appropriate channels, i.e. social media, lay conferences, public engagement events, service provider events, and newsletter articles. Lay partners also sit on the trial steering committee (TSC) as verified independent members and are supported by the Chief Investigator (CI), trial management team, and through the peer support of lay partners on existing clinical trials.

### Trial design {12}

**Fig. 1:**
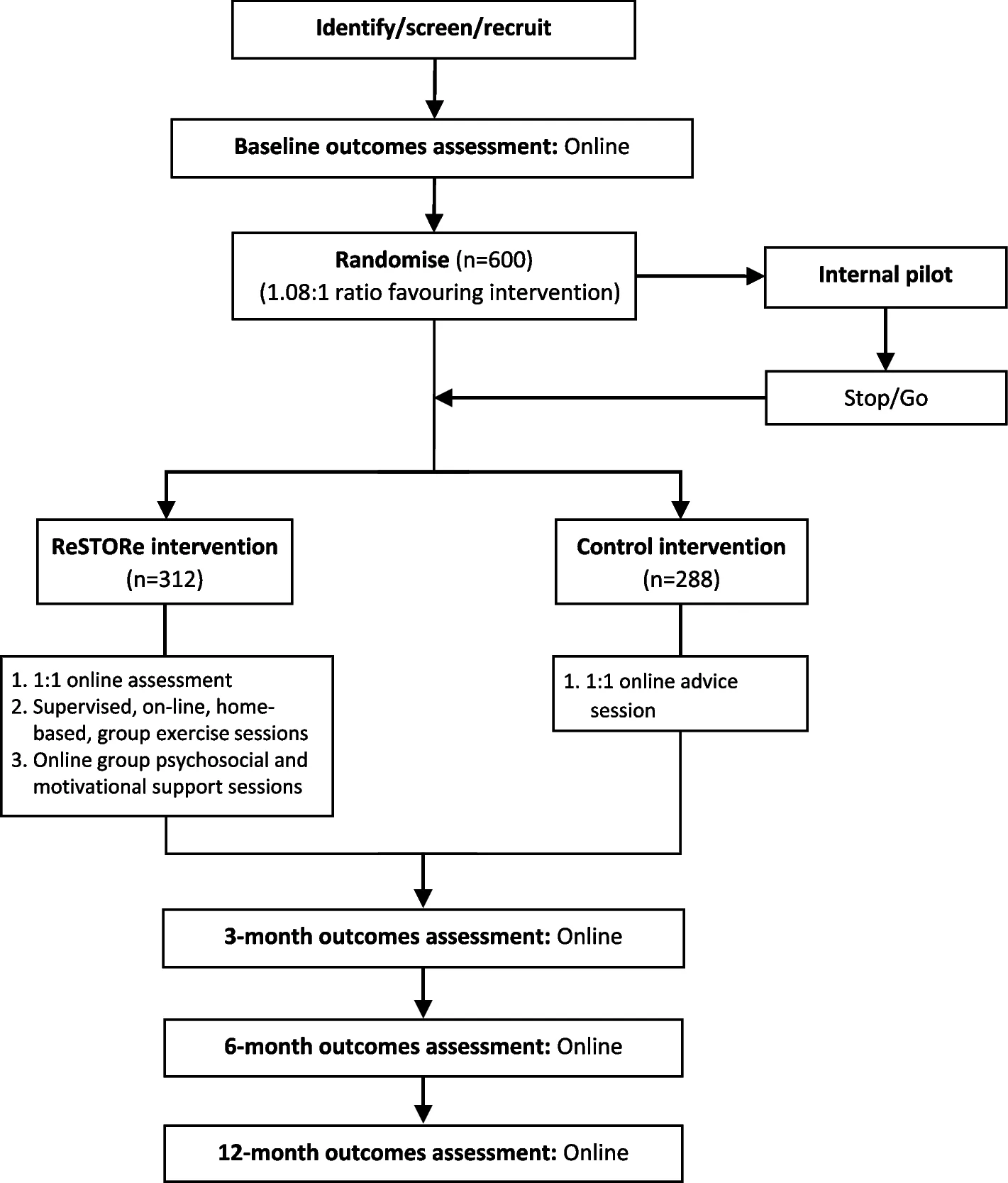
ReSTORe flow chart

ReSTORe is a pragmatic, parallel group, superiority, multi-centre, decentralised, two-arm RCT with an internal pilot phase and embedded economic evaluation and qualitative study (Fig. 1).

#### Pre-pilot

We followed MRC guidance for the development of complex interventions [13]. Through systematic review of the literature, expert opinion, and stakeholder engagement and consensus meetings, we identified existing exercise-based rehabilitation intervention components suitable for adaptation and refinement for testing with people recovering from stroke, many of whom may have communication impairments. Data from our systematic review [5], co-designed stroke exercise programme [14], portfolio of existing online rehabilitation interventions [15–17], and long-standing stroke service user group and qualitative research [18] provided a strong evidence base with which to develop and refine the ReSTORe intervention. During the 6-month set-up/pre-pilot period, we finalised the intervention and trial procedures for stroke survivors, specifically adapting procedures and materials for those with communication impairments. The refined ReSTORe programme, including the participant and practitioner manuals, was tested in a pre-pilot with seven participants, along with testing control materials, conducting staff training, and preparing trial set-up. The development of the ReSTORe intervention will be described in full in future publications.

#### Internal pilot

During months one to three (from first randomisation), we aim to enrol ~64 participants (~four/five intervention groups). In months 4 to 6, we aim to recruit ~45 participants/month (~three/four intervention groups) before progressing seamlessly to the main trial. If achieving less than 50% of our targets, we will consider recruitment unlikely to be feasible; if 50 to 99%, the trial management group (TMG) will review recruitment strategies, report to the trial steering committee (TSC), data monitoring committee (DMC), and funder, and continue with a modified protocol and close monitoring.

## Methods: participants, interventions, and outcomes

### Trial setting {13}

The trial will recruit from primary and secondary care participant identification centres (PICs) in England, Scotland, and Wales. Interventions will be delivered online from a central trial hub to participants in their homes.

### Characteristics of the people who are needed for the trial

**Table:** 

Characteristic	The people we would expect to see included
Age	Median 76 (inter-quartile range 65 to 84)
Sex	Male: 54.1% Female: 45.8%
Gender	Male: 54.1% Female: 45.8% Other: 0.1%
Race, ethnicity, and ancestry	White: 80% Black: 3% Asian: 5% Other: 12%
Socioeconomic status	Unknown
Geographic location	England: 80% Scotland: 10% Wales: 10%
Other characteristics relevant to the trial	Not applicable

### Eligibility criteria for participants {14a}

Adults with long-term mild to moderate physical and/or mental health disability after stroke are eligible to participate if they meet the trial inclusion criteria (Table 1).

**Table 1:** Inclusion and exclusion criteria

Inclusion criteria	Exclusion criteria
▪ UK resident ▪ Aged ≥18 ▪ 6 to 36 months post-hospital discharge after admission for stroke ▪ Participant self-report of ongoing post-stroke physical and/or mental health problem(s) ▪ Simplified modified Rankin Scale questionnaire (smRSq) score ≤4 [19] ▪ Language and cognitive ability sufficient to follow visual instruction and complete study activities, with support as required ▪ Access to, and ability/support to use, a computer or device with internet audio and video ▪ Able to understand spoken and written English themselves, or with support from family or friends	▪ Exercise contra-indicated based on internationally accepted guidelines [20] ▪ Severe physical disability (smRSq > 4) or inability to sit or stand independently with or without aid ▪ Mental health or cognitive impairment sufficient to prevent engagement with the study or make participation unsafe ▪ Living in locations other than ‘home’, e.g. prison, nursing home, and intermediate care

### Eligibility criteria for sites and those delivering interventions {14b}

Participants will be identified via several routes (sites) including (1) primary care—Regional Research Delivery Networks (RRDNs), (2) secondary care—NHS Digital Digi-Trials recruitment service, (3) participant databases (e.g. National Institute for Health Research voluntary ‘Be Part of Research’ register), and (4) self-referral. Database searches will identify potential participants according to pre-specified inclusion criteria.

### Who will take informed consent? {32a}

Eligible participants will complete informed consent and baseline outcome measures online and will subsequently be randomised electronically.

### Additional consent provisions for collection and use of participant data and biological specimens {32b}

Not applicable—no additional consent provisions required. Consent for all study activities will be obtained during the informed consent process.

## Intervention and comparator

### Intervention and comparator description {15a}

#### ReSTORe intervention

The ReSTORe multi-component rehabilitation intervention is designed to improve post-stroke fatigue, physical and mental health, and HRQoL. Whilst not designed specifically to improve communication and cognitive impairments, all trial procedures and materials are adapted to improve accessibility for people with communication impairments such as aphasia who are commonly excluded from rehabilitation research. The ReSTORe intervention is a 10-week supervised, live online, home-based, group rehabilitation programme with three key components:

1. Individual assessment: 1-h, online, one-to-one assessment with a ReSTORe practitioner (clinical exercise physiologist or physiotherapist), trained and supported by a health psychologist and stroke communication specialist (speech and language therapist), to screen, assess, and provide personalised rehabilitation advice.
2. Exercise: Up to 45 min, 2–3 times/week for 10 weeks consisting of personalised, individualised, multi-modality, equipment-free (or minimal equipment) aerobic and resistance exercise at a manageable intensity. The exercise component is optimised to be appropriate for a broad spectrum of participants including frailer, deconditioned (including those with spasticity), low-mobility and exercise-naive participants. Exercise intensity and duration will be progressed in an individualised fashion in response to participant feedback. In line with patient partner preferences, the ReSTORe exercise intervention includes:
  - Live (end-to-end encrypted, password-protected) online group sessions led by ReSTORe practitioners to allow group exercise with real-time instruction and feedback. Groups will have around eight participants and will be age, ability, culturally, and communication impairment specific in smaller discrete groups as required.
  - Signposting to additional pre-recorded on-demand sessions, graded by ability, including a range of modalities from breathing exercises to chair-based and standing moderate and high-intensity functional exercise.
  - Participant workbook with instructions on safe and effective exercise.
3. Psychosocial and motivational support: Participants will attend six online 45–60-min group support sessions facilitated by a ReSTORe practitioner, with training and support from a Health Psychologist. Sessions will cover the cognitive and emotional impact of living with stroke and support engagement with the exercise component of the intervention by addressing potential barriers such as fear avoidance of activity and fatigue. The intervention is underpinned by behaviour change theory and designed in accordance with complex intervention (MRC/NIHR) frameworks. We also draw on guidance for designing behaviour change interventions based on the target population, target behaviours, hypothesised mechanism of change, and behaviour change technique [21]. The concepts underpinning the development of these sessions have been demonstrated to have beneficial effects on mental well-being in people with stroke [22]. The six sessions are (1) motivation and goal setting, (2) avoidance of activity, (3) managing post-stroke fatigue, (4) mood and emotions, (5) stress and stress management, and (6) managing setbacks, long-term change, and future goals.

Delivery: The intervention will be delivered UK-wide from a single trial hub (an approved NHS rehabilitation centre in Coventry). Practitioners delivering the ReSTORe intervention will be clinical exercise physiologists or physiotherapists with specialist exercise rehabilitation qualifications, appropriate professional registration, relevant continued professional development, and good clinical practice (GCP) training. This centralised remote delivery model allows concentration of professional expertise and experience, standardised delivery, and time and resource-efficient trial and intervention procedures. For participants, decentralised online delivery supports accessibility for those who would otherwise not be able to take part in centre-based rehabilitation programmes due to poor health, cost, transport, and time pressures. People with communication impairments will be accommodated in smaller discrete groups, practitioners will be trained by stroke communication specialists, and a ‘co-pilot’ (additional practitioner) will provide technical assistance and monitor participant safety. People who do not have access to a suitable electronic device will be provided with one.

Safety: Sessions will be led by staff experienced in remote online assessment, prescription, and delivery of physical activity for multi-morbid clinical populations. Troubleshooting and screening before and during live exercise and support sessions will assess safety, progress, health, and any adverse effects. Participants will be advised to initially have another person nearby whilst exercising. A comprehensive emergency procedure has been developed and implemented in existing trials, ensuring, in the unlikely event of someone becoming unwell during live sessions, protocols are in place to efficiently manage the situation.

Training: ReSTORe practitioners will be upskilled in stroke communication with training delivered by specialist stroke speech and language therapy professionals, with psychosocial, motivational, and behavioural support training delivered by experts in health psychology. Practitioners will undergo a single day of ReSTORe training to ensure an appropriate level of clinical knowledge and skills for exercise-based rehabilitation in long-term stroke survivors. The training will be supported by a comprehensive practitioner manual.

#### Control intervention

A single 1:1 ReSTORe practitioner appointment, with general advice on safe and effective physical activity and living with stroke using freely available Stroke Association leaflets (https://www.stroke.org.uk/getting_active_after_stroke_guide.pdf) including aphasia-friendly and translated versions. A 30-min 1:1 online appointment will allow the practitioner to discuss individualised ways in which the participant can independently engage in self-directed physical activity. Participants will not be provided with a structured exercise plan; rather information suggesting ways in which physical activity can be incorporated into their everyday lives. No specific psychological techniques will be used in providing this information.

### Criteria for discontinuing or modifying allocated intervention/comparator {15b}

Participants may decline to continue involvement in the study at any time, without prejudice. Participants can discontinue from the trial intervention (treatment discontinuation), and they can also choose to withdraw from the trial completely. This will not affect the standard of care they receive. For participants withdrawing from the study altogether, data obtained prior to the point of withdrawal will be retained for the final analysis unless explicitly withdrawn at the participant’s request.

### Strategies to improve adherence to intervention/comparator {15c}

Intervention participants will attend six online 45–60-min group support sessions facilitated by a ReSTORe practitioner, to support engagement with the exercise component of the intervention by addressing potential barriers such as fear avoidance of activity and fatigue. Participants will be contacted by email and telephone if they do not attend scheduled sessions.

### Concomitant care permitted or prohibited during the trial {15d}

All concomitant care and interventions are permitted.

### Ancillary and post-trial care {34}

There is no provision for ancillary and post-trial care. Incidental findings relating to participants’ medical conditions or general health will be discussed with the participant’s GP and communicated to the participant as required. The participant will be advised to make an appointment with their GP to discuss the findings. The sponsor provides cover in case something goes wrong. If participants are harmed by taking part, compensation may be available. If the harm was caused by someone’s mistake, legal action may be appropriate. Independent advice is provided by the Patient Advice and Liaison Service (PALS).

### Outcomes {16}

#### Primary outcome

We will use the Patient-Reported Outcomes Measurement Information System (PROMIS) 29+2 Profile v2.1 [23] to generate our primary outcome and a number of relevant secondary outcomes. The PROMIS 29+2 Profile is one of a portfolio of outcomes from the USA National Institute for Health (NIH). It is reliable, generic, and validated for online use. The primary outcome is health-related quality of life (HRQoL) at 6 months post-randomisation using the PROMIS-Preference Score (PROPr). The PROPr score is generated from seven sub-scores: depression, fatigue, sleep disturbance, pain interference, physical function, social roles/activities, and cognitive function [24, 25]. The score ranges from −0.022 to 1.0 where 0 indicates a health state equivalent to death and 1.0 indicates perfect health [23]. The mean PROPr score will be compared between the control and intervention groups to estimate the intervention effect.

#### Secondary outcomes

A further two PROMIS sub-scales will independently measure anxiety and pain intensity. All PROMIS sub-scores and sub-scales are rated over the preceding 7 days apart from physical function and social roles/activities which do not have a specified timeframe. For analysis purposes, the sub-scale scores are converted to standardised *T*-scores with a mean of 50 and a standard deviation (SD) of 10. Higher *T*-scores represent more of the concept being measured. For anxiety, depression, fatigue, pain interference, and sleep disturbance, higher scores indicate more severe symptoms. For physical function and social participation, higher scores indicate better health outcomes. Secondary outcomes are:

1. HRQoL: PROMIS 29+2 Profile.
  - PROPr score (3 and 12 months) (mean [SD]).
  - Sub-scores: depression, fatigue, sleep disturbance, pain interference, physical function, social roles/activities, and cognitive function [23, 25] (mean [SD]).
  - Sub-scales: anxiety and pain intensity (mean [SD]).
2. Cognitive function: PROMIS Neuro-QoL short-form v2.0. An 8-item neurological condition specific assessment of cognitive function [26] (mean [SD]).
3. Health utility: EQ-5D-5L score, a validated, generic HRQoL measure consisting of five dimensions, each with five levels of response. Each combination of answers can be converted into a health utility score. It has good test-retest reliability, is simple to use, and gives a single preference-based index value of health status for cost-effectiveness analysis [27] (mean [SD]).
4. Physical activity: International Physical Activity Questionnaire Short-Form (IPAQ-SF), a measurement of both volume and intensity of physical activity participation [28] (categorical [count, %]).
5. General health: self-report of current overall health compared to baseline (categorical [count, %]).
6. Health and social care resource use: participant self-report.
7. Cerebrovascular morbidity: whether a participant has recurrent stroke (categorical [count, %]).
8. All-cause and cardiovascular mortality (categorical [count, %]).
9. Safety: total adverse events attributable to the trial (count).
10. Safety: total serious adverse events (count).

Follow-up: Outcomes will be collected online pre-randomisation and at 3, 6, and 12 months post-randomisation. In the case of non-response to online questionnaires, we will seek to collect outcomes by telephone, specifically two key outcomes (PROPr and EQ-5D-5L) if time is limited. Fluency in written English is not an inclusion criterion for this study. For those able to speak and understand spoken English, but not written English, we will collect PROPr and EQ-5D-5L verbally. Both questionnaires are well validated for verbal administration. This approach reduces the exclusion of the most socially disadvantaged and those who do not speak English as their first language. We will also provide support in completing outcomes assessments for people with stroke-related communication impairments.

### Harms {17}

From randomisation to the 6-month primary outcome timepoint, adverse events (AE) and serious adverse events (SAE) will be recorded in both trial arms, in line with the principles of good clinical practice [29], and identified via participant and next-of-kin self-report at outcomes assessment time points, intervention sessions, or by contacting the trial team directly. An AE will be defined as any untoward medical occurrence in a participant which does not necessarily have a causal relationship with the intervention. Adverse events exempt from formal recording include participants’ normal post-stroke symptoms and sequelae, normal post-exercise symptoms, and exacerbation of pre-existing musculoskeletal conditions lasting less than 72 h. These will be documented in participant notes for clinical purposes only. Any AEs outside of these criteria, and related to the ReSTORe trial, will be recorded in accordance with local protocol, i.e. recorded on the trial database using an AE form. Adverse events that are deemed to be serious (SAE) will be reported to the WCTU trial team and assessed for causality by the CI and another appropriately delegated clinical trial team member. Serious adverse events that have no causal relationship with the intervention will not be reported to oversight committees. Those deemed to be unexpected and possibly, probably, or definitely related to the trial interventions or procedures will be notified to the trial sponsor and the Research Ethics Committee (REC) within 15 days. All AEs and SAEs will be recorded within 24 h of the investigator being made aware. In supplementary material, SAEs will be classified using Common Terminology Criteria for Adverse Events (CTCAE).

### Participant timeline {18}

**Table:** 

Assessment time point	Pre-randomisation		Post-randomisation			
	1	2	3	4	5	6
	Screening	Enrolment/randomise (baseline)	Intervention delivery 10 weeks	3 months (±2 weeks)	6 months (±1 months)	12 months (±1 months)
Invitation	X					
Initial screening check	X					
Eligibility assessed	X					
Concomitant illnesses		X				
Additional screening check (telephone if required)		X				
Informed consent		X				
Demographics		X				
Intervention delivery			X			
Health and medication use		X		X	X	X
Simplified modified Rankin Scale		X		X	X	X
PROMIS-PROPr		X		X	X	X
PROMIS® 29+2 Profile v2.1		X		X	X	X
PROMIS Neuro-QoL		X		X	X	X
EQ-5D-5L		X		X	X	X
IPAQ-SF		X		X	X	X
General health (self-report)		X		X	X	X
Recurrent stroke		X		X	X	X
Adverse events			X	X	X	X
Death			X	X	X	X
Health and social care resource use				X	X	X
Semi-structured interviews (process evaluation)				X		

### Sample size {19}

To account for unequal variances between groups due to clustering in the intervention group, we shall utilise a partial cluster randomisation design and Moerbeek’s method [30] (allocation ratio 1.08:1), thus needing to recruit *n* = 600 (intervention *n* = 312; control *n* = 288) participants to detect a target difference of 0.04 on the PROPr score, with a standard deviation of 0.18 (data from the REGAIN trial) [10, 31], a mean cluster size in the intervention arm at randomisation of 8, a significance of 5%, power of 90%, and 20% loss to follow-up. An intra-class correlation coefficient (ICC) of 0.03 and a correlation for the primary outcome between baseline and 3 months of 0.7 (lower limit of 95% confidence interval, CI) [32] are assumed based on data from the REGAIN trial.

### Recruitment {20}

Large volume invitation postal mailout will be conducted in batches over 15 months. Potential participants will visit an online portal to access a short video explainer and complete a suitability check (simple yes/no questions) and eligibility check. Eligible participants will be prompted to complete informed consent and baseline outcome measures online and will subsequently be randomised electronically. At the expression of interest stage, people with communication impairments can request support from a practitioner trained in stroke communication. These people will be contacted by audio/video called by a clinical team member to confirm eligibility using an adapted Consent Support Tool communication screening test as required [33] and to guide them through consent and outcomes assessment procedures.

## Assignment of interventions: randomisation

### Sequence generation: who will generate the sequence {21a}

Computer-generated randomisation sequence administered by the Warwick Clinical Trials Unit (WCTU) centralised service.

### Sequence generation: type of randomisation {21b}

Randomisation will be performed in a ratio of 1.08:1 (intervention vs. control) by minimisation with sex, age (≥ or <65 years), and simplified modified Rankin Scale (≤2 or 3–4) score as stratification variables.

### Allocation concealment mechanism {22}

All baseline data will be collected before randomisation to the ReSTORe intervention or best-practice usual care. Allocation will be concealed from some members of the trial team and the trial management group (TMG). In the event participants need to be contacted directly, for example to collect missing outcome data, we will request not to be informed of treatment allocation.

### Implementation {23}

Electronic randomisation.

## Assignment of interventions: blinding

### Who will be blinded {24a}

It will not be possible to blind participants or intervention practitioners. Allocation will be concealed from some members of the trial team.

### How will blinding be achieved {24b}

Not applicable. It will not be possible to blind participants or intervention practitioners.

### Procedure for unblinding if needed {24c}

Due to the nature of the intervention, participants and those delivering the intervention will be aware of the treatment allocation. By virtue of the design, it is not possible to blind participants, intervention practitioners, or the site researchers. The TMG will be blinded to group allocation until data analysis is complete. Therefore, there is no requirement for unblinding procedures due to the open-label nature of the trial.

## Data collection and management

### Plans for assessment and collection of outcomes {25a}

All outcomes are patient reported and will be collected online pre-randomisation and at 3, 6, and 12 months post-randomisation.

### Plans to promote participant retention and complete follow-up {25b}

An email with the link to the follow-up questionnaires will be emailed to participants at each outcome timepoint. Several reminders will be sent. In the case of non-response to online questionnaires, we will seek to collect outcomes by telephone, specifically two key outcomes (PROPr and EQ-5D-5L) if time is limited.

### Data management {26}

Personal data collected during the study will be handled and stored in accordance with the UK General Data Protection Regulation (GDPR). Personal identifying information will be collected via an online database and stored electronically at WCTU within a bespoke Clinical Data Management System (CDMS). For quality assurance, the data and results will be routinely checked for completeness and accuracy. Trial documentation and data will be archived for 5 (UoW) or 10 (UHCW) years after completion of the trial.

### Confidentiality {33}

All essential documentation and trial records will be stored in conformance with the applicable regulatory requirements and access restricted to authorised personnel. All data will be pseudonymised after collection of baseline demographics. Handling of personal and confidential data will be clearly documented in the participant information sheet and consent obtained. ReSTORe specialists at UHCW NHS Trust (sponsor) will keep paper records of participant contact details and medical health information for those participants randomised to the ReSTORe intervention. This is required for study delivery to ensure participants are exercising at an appropriate level and to be used if a medical emergency occurs. These paper records will be stored securely in locked filing cabinets only accessible to study staff.

## Statistical methods

### Statistical methods for primary and secondary outcomes {27a}

Trial results will be reported in accordance with Consolidation Standards of Reporting Trials (CONSORT) guidelines. Primary analyses will be intention to treat using a partially nested heteroscedastic regression model to estimate the intervention effect (with 95% CIs), adjusted for clinically important variables. This is to accommodate for the clustering in the intervention group and the individual patient observation in the control arm. We will examine the clustering effect and, if this is significant, we will fit the above models with the clustering variable as a random effect; otherwise, it will be fitted as a fixed effect. We will use an estimand framework to assess intercurrent events (ICEs) and handle any missing data. Intercurrent events will include non-compliance (as defined by criteria agreed with the oversight committees), assessed using CACE (Complier Average Causal Effect) analysis [34]. Continuous secondary outcomes will be fitted in a similar way to the primary outcome. For categorical outcomes, analogous models will be computed [35] with summaries based on odds ratio and 95% CI of odds ratios. Withdrawal and adverse event data will be compared between the arms using chi-squared test statistics. Graphical plots will be produced displaying the means and CI from baseline over the three follow-up periods. A pre-specified statistical analysis plan will be approved by the DMC and TSC.

### Who will be included in each analysis {27b}

All analysis will be done using treatment policy strategy, i.e. intention-to-treat (ITT). The participants will be analysed according to the treatment arm they were allocated to, irrespective of the treatment they received. All participants will be included in the analysis regardless of whether they adhered to the protocol.

### How missing data will be handled in the analysis {27c}

If the proportion of missing outcome data is less than 5% then only complete case analysis will be performed (i.e. excluding cases with missing outcome data). If the proportion of missing outcome data is above 5%, multiple imputation method will be considered under the assumption that data are missing at random (MAR).

### Methods for additional analyses (e.g. subgroup analyses) {27d}

The following subgroups will be considered:

1. Age (<65 vs. ≥65 years)
2. Sex (male vs. female)
3. Simplified modified Rankin Scale (≤2 vs. 3–4)

These subgroup analyses will be performed on the ITT strategy. The primary outcome will be used as the dependent variable, and the interaction between treatment and subgroup will be considered. Linear regression models will be used to assess the subgroup effect, using interaction terms. As these analyses are post hoc analyses which are not powered for any effect size, emphasis will not be based on the statistical testing, rather than the point estimates and 99% confidence intervals.

### Interim analyses {28b}

Not applicable.

### Protocol and statistical analysis plan {5}

This will be published separately to this submission and will be available on the trial website: https://warwick.ac.uk/fac/sci/med/research/ctu/trials/restore/.

### Health economic evaluation and analysis

We will conduct a prospectively planned within-trial cost-utility analysis with a 12-month time horizon from an NHS and personal social services perspective. Patient-level resource use data will be captured 3, 6, and 12 months after randomisation via online resource use questionnaires (RUQs). These data will comprise the following categories: inpatient admissions (e.g. length of hospital stay, hospital ward); outpatient appointments (e.g. reason); accident and emergency attendance (e.g. reason); primary and community care services (e.g. number of visits, average time spent per visit); any social services, equipment/aids; and new prescribed medications provided by NHS, and will be costed using the most recent UK national reference costs, reflated to a common year. Data will also be collected during delivery of the intervention sessions including the expertise and grade of practitioners, the duration of sessions delivered, the number of attendees, and the materials and equipment used to deliver the intervention. These data will be used to derive a unit cost per participant for the intervention, based on reference costs and NHS mid-point pay scales. Capital costs will be amortised over their expected lifetimes.

Generic HRQoL will be assessed at baseline, 3, 6, and 12 months using an online EQ-5D-5L questionnaire. If participants do not complete online questionnaires, they will be contacted by phone. EQ-5D-5L scores will be converted to health status scores using the UK value set recommended by NICE guidance at the time of analysis [36]. Patient-level quality adjusted life year (QALY) estimates will be calculated using the trapezoidal rule using utilities generated via the EQ-5D-5L in surviving patients.

Selected statistical methods will deal with skew, baseline imbalance, missingness [37, 38], and sampling uncertainty, as appropriate. Every effort will be made to minimise missingness. If missingness of patient-level costs or QALYs is ≤5%, the primary analysis will use complete case data. If missingness exceeds 5%, mechanisms will be explored and multiple imputation methods applied [39]. We will use bootstrapped bivariate analyses of costs and QALYs to generate within-trial incremental cost per QALY estimates and confidence intervals. Findings will be analysed and visualised as cost-effectiveness acceptability curves (CEACs) [40] using the net-benefit approach to show the probability that ReSTORe is the optimal choice over a range of possible values of the ceiling ratio. Sensitivity analysis will explore the impact of broadening the decision perspective beyond the NICE reference case to include indirect costs such as the time lost from work.

If findings are non-convergent at 12 months, we will explore the sensitivity of cost-effectiveness to extrapolation of costs and benefits beyond the trial time horizon, via a suitable decision model or parametric survival analysis model or extrapolation of net monetary benefit. We will also conduct a secondary cost-effectiveness analysis to derive the unit cost of improvement in the primary outcome (PROPr scores). Details of the prospective plan and analysis will be described in detail in the Health Economics Analysis Plan (HEAP) written by the trial health economists in line with guidance from Warwick CTU standard operating procedure (SOP) 21. Reporting will follow the Consolidated Health Economic Evaluation Reporting Standards (CHEERS) statement [41].

### Embedded process evaluation

The aims of the process evaluation are three-fold: (1) to understand the experiences of the intervention, including enablers and barriers; (2) to explore the implementation of the intervention, including fidelity; (3) to identify any contextual factors that impact on the study and/or the interventions. At the consent stage, participants will have the option to indicate that they are happy to be contacted with regard to completing a semi-structured interview with a researcher. Later, participants will be approached by email or text if they agreed to this on their main trial consent form. We will send a link to a participant information sheet, and if they wish to take part, a consent form, returned electronically to the researcher. All interviews will be carried out remotely using MS Teams and will be recorded, transcribed, and anonymised by an approved transcription service. Support from stroke communication experts will be available as required. All data will be held in compliance with GDPR requirements and be organised and explored with NVivo software.

In the internal pilot, participant interviews will assess experiences and intervention acceptability and trial procedures (*n* ~ 5 per arm), conducted at around 3 months post-randomisation. These interviews will be used to optimise the subsequent recruitment, retention, and intervention design of the main study. In the main study, the embedded process evaluation will target understanding participant and practitioner experiences. We will undertake qualitative interviews with up to 25 participants in each arm to understand experiences and intervention-related barriers and enablers. Between 16 and 24 interviews are recommended to reach saturation of meaning [42]. Purposive sampling, including gender, ethnicity, and age, will be utilised to ensure a diverse range of views are gathered. We will undertake the participant interviews after the primary outcome data has been collected 6 months post-randomisation. Healthcare professionals delivering the intervention will a be interviewed after their last intervention group or best-practice usual care session (*n* ~ 8). Questions on the credibility of the intervention and expectancy of success and self-efficacy will be collected at baseline and 6 months after randomisation. Interviews will be analysed using framework analysis [43], to help us understand what happened during the study and if and how participants used the interventions. Further thematic analysis [44] will explore barriers and enablers and participants’ experiences of their recovery, as well as healthcare professionals’ experiences of delivering the ReSTORe and control interventions.

To understand the fidelity of intervention implementation, we will assess practitioner delivery competence. Fidelity using adherence and competence will be scored for each step/aspect (2 evident, 1 partially evident, 0 not evident, and N/A not appropriate) and an overall average given. We will generate total averages with ranges for adherence and competence. This will be undertaken by audio recording all control and intervention sessions and reviewing a 10% sample using predetermined checklists to confirm compliance with the procedures and trial information. In addition, 10% of these assessments will be double coded.

## Oversight and monitoring

### Composition of the coordinating centre and trial steering committee {3d}

The TMG, consisting of project staff, co-investigators, and ‘lay’ representatives involved in the day-to-day running of the trial, will meet monthly throughout the project. Any significant issues will be referred to the TSC which will meet twice yearly and constitute a group of experienced trial staff, clinicians, academics, trialists, ‘lay’ representatives, and an independent chairperson.

### Composition of the data monitoring committee, its role and reporting structure {28a}

A data monitoring committee (DMC) consisting of independent experts with relevant clinical research experience will meet twice yearly to ensure data integrity and participant safety.

### Frequency and plans for auditing trial conduct {29}

The study will be monitored by the Research and Development department at UHCW as representatives of the lead sponsor (the monitor will be appropriately trained in accordance with local standard operating procedures, SOP) and by the Quality Assurance team at WCTU as representatives of the study coordinating centre and academic lead, to ensure that the study is being conducted as per protocol, adhering to Research Governance and GCP. The approach to, and extent of, monitoring will be specified in a study monitoring plan determined by the risk assessment undertaken prior to the start of the study. A Trial Monitoring Plan will be developed and agreed by the TMG and TSC based on the study risk assessment, including on-site monitoring if applicable. Processes to be considered in the monitoring plan will include participant enrolment, consent, eligibility, and allocation to study groups; adherence to study interventions and policies to protect participants, including reporting of harm and completeness, accuracy, and timeliness of data collection. The plan will be available from the study coordination centre and will also be lodged with the sponsor. Whilst the monitors work in the same institution as the study team (WCTU), they will act independently in this role. If UHCW has evidence that the study protocol and procedures are not being adhered to, an on-site monitoring visit may be triggered.

### Protocol amendments {31}

Substantial protocol amendments (e.g. changes to eligibility criteria) will be communicated in writing (email) by the study team to all relevant parties, e.g. investigators, participants, NHS Trusts, and study registries once approved.

### Dissemination policy {8}

Full results of the study will be prepared by the research team and lay partners and submitted to funders as a final report. Findings will be submitted to peer-reviewed journals and disseminated to the medical and rehabilitation communities. We will publish papers in open-access journals describing the development and refinement of the ReSTORe intervention, and the study protocol, as per recommended guidance for transparent reporting, the Consolidated Standards of Reporting Trials (CONSORT) guidelines. We will submit abstracts to national and international conferences. If appropriate, the ReSTORe intervention will be manualised and available for public access once the study has completed, and we will develop a practitioner training programme to support the implementation of ReSTORe. Our lay partners will help prepare the final report and assist with dissemination of study results. We will produce a lay summary for participants and the hospitals/centres involved. Results will be publicised via the study website. The ReSTORe study will be relevant to the NHS; thus, outputs will follow the usual route into the NHS system and wider society.

## Discussion

Long-term mild to moderate disability including fatigue, low functional capacity, muscle weakness, anxiety, and depression is relatively common after a stroke [1] leading to reduced HRQoL [3]. Ongoing physical and mental health support for this group is limited. Exercise-based rehabilitation programmes, conventionally delivered face-to-face in rehabilitation centres, may help people after stroke, but are rarely provided in the UK. As an alternative, group rehabilitation delivered online has proven feasible and acceptable to people with other long-term conditions [7, 8] and may be of benefit to people recovering from stroke. The ReSTORe trial aims to test the clinical and cost-effectiveness of supervised, live online, home-based, group exercise-based rehabilitation to support long-term physical and mental health recovery versus best-practice usual care, for people with mild to moderate disability, 6–36 months post-hospital discharge after stroke. The trial will be the largest study undertaken to date exploring online exercise rehabilitation for people recovering from stroke. Results from a robustly conducted RCT with economic evaluation and embedded process evaluation will provide high-quality evidence to inform guideline development for stroke rehabilitation services in the UK NHS and beyond. People with communication impairments after stroke are often excluded from clinical trials testing rehabilitation programmes. The ReSTORe trial has undertaken a detailed and thorough development process with patient representatives and specialist communication experts to ensure improved accessibility for this group, thus helping to ensure a representative sample. The decentralised approach to patient identification, recruitment, and delivery will also facilitate greater control over inclusivity and diversity by helping to ensure productive engagement with underrepresented populations.

## Trial status

Recruitment began in April 2025 and is expected to complete in May 2026.

## Supplementary Information

Supplementary Material 1.

Supplementary Material 2.

## Data Availability

All data requests should be submitted to WCTUDataAccess@warwick.ac.uk for consideration. Access to anonymised data may be granted following review.

## Abbreviations

AE: Adverse event
CACE: Complier Average Causal Effect analysis
CI: Confidence interval
CDMS: Clinical Data Management System
CONSORT: Consolidation Standards of Reporting Trials
CEAC: Cost-effectiveness acceptability curve
COVID-19: Coronavirus disease 2019
CHEERS: Consolidated Health Economic Evaluation Reporting Standards
DMC: Data Monitoring Committee
EQ-5D-5L: EuroQol 5-Dimension 5-Level
GCP: Good clinical practice
GDPR: General Data Protection Regulation
HEAP: Health Economics Analysis Plan
HRQoL: Health-related quality of life
ICC: Intra-class correlation coefficient
ICE: Intercurrent event
IPAQ-SF: International Physical Activity Questionnaire Short-Form
ISRCTN: International Standard Randomised Controlled Trial Number
MRC: Medical Research Council
NICE: National Institute for Health and Care Excellence
NHS: National Health Service
NIH: National Institute for Health
NIHR: National Institute for Health Research
PIC: Participant identification centre
PROMIS: Patient-Reported Outcomes Measurement Information System
PROPr: PROMIS-Preference Score
QALY: Patient-level quality adjusted life year
RCT: Randomised controlled trial
REC: Research Ethics Committee
ReGAIN: Rehabilitation exercise and psychological support after COVID-19 infection
ReSTORe: Remote STrOke Rehabilitation
RRDN: Regional Research Delivery Network
RUQ: Resource use questionnaire
SAE: Serious adverse event
SD: Standard deviation
SMD: Standardised mean difference
smRSq: Simplified modified Rankin Scale questionnaire
SPIRIT: Standard Protocol Items, Recommendations for Interventional Trials
SSNAP: Sentinel Stroke National Audit Programme
SOP: Standard operating procedure
TMG: Trial management group
TSC: Trial steering committee
UHCW: University Hospitals Coventry and Warwickshire NHS Trust
UK: United Kingdom
WCTU: Warwick Clinical Trials Unit
