# Supplementary material for: Remote STrOke Rehabilitation (ReSTORe): study protocol for a randomised controlled trial
Source: Trials. 2026 Aug 13;27:657. doi: 10.1186/s13063-026-09952-5 (PMC13749901; doi:10.1186/s13063-026-09952-5)
Supplement: Supplementary file 1 [file 13063_2026_9952_MOESM1_ESM.pdf]

NIHR | National Institute for Health and Care Research

NIHR Coordinating Centre
Alpha House
Enterprise Road
Southampton
SO16 7NS

Email: htacommissioning@nihr.ac.uk

Dr Gordon McGregor
Associate Clinical Professor
University Hospitals Coventry & Warwickshire NHS Trust
University Hospital
Clifford Bridge Road
Coventry
England
CV2 2DX

16 October 2023

Dear Dr McGregor

Reference: NIHR157510 Remote STrOke Rehabilitation (ReSTORe): a UK-wide randomised controlled trial with embedded process evaluation and economic analysis.

I am pleased to inform you that an HTA Funding Committee has recommended your application submitted for consideration in the call: **22/166 Rehabilitation and care following stroke** for funding. The Department of Health and Social Care in its capacity as the National Institute for Health and Care Research (NIHR), has confirmed its intention to award funding upon acceptance of the terms and conditions set out in the Standard Research Contract and pending agreement to the suggested amendments recommended by the Funding Committee, as detailed in the enclosures.

The Standard Research Contract, between Contractors and the Secretary of State for Health and Social Care for all initiatives can be found on the NIHR website at:

https://www.nihr.ac.uk/researchers/apply-for-funding/how-to-apply-for-project-funding/sign-a-contract.htm.

Next Steps

The NIHR is committed to the rapid initiation of research following the decision to fund to benefit members of the public and patients as soon as possible. Therefore, we expect funded researchers to be working towards gaining the necessary contractual agreements and governance approvals required to start the project within 8 months or by a date mutually agreed by both parties on acceptance of the award.

The NIHR acknowledges the risk to organisations around committing resource to research before a contract is in place; however, it is rare to not reach contractual terms unless the circumstance of the research team changes. The NIHR, therefore, encourages organisations to commit staff to setting up projects at as early an opportunity as possible in order to expedite the formal commencement of research.

It is acknowledged that there can be unforeseen delays in starting up a research project, but in order to help reduce these it is your responsibility to work closely with your organisation's

R&D department or equivalent as well as other colleagues/departments involved in the administration and management of the research, and to start these discussions at the earliest opportunity.

To ensure that the project starts within the agreed timeframe with all the required agreements and approvals in place, appropriate staff (such as project and/or study managers) need to be in post as early as possible after receiving this letter of intent. These staff costs will ultimately be covered through the research funding award, but you are encouraged to meet them from Research Capability Funding (RCF) or equivalent prior to the research contract being agreed.

To support the often-iterative process towards agreement of the contract, we have set out the timeframes for the submission of responses or information for each step towards the agreement of the Standard Research Contract as well as the anticipated start date.

- Responses to Funding Committee feedback and queries – Four weeks from the date this email notification was sent
- Responses to Finance and IP queries – Four weeks from the date this email notification was sent
- Contract signature – no later than 1 month from the date the contract is issued for signature.
- Contracted commencement start – no later than 8 months from the date this email notification was sent or a date mutually agreed on acceptance of award.

On receipt of information as set out above, the NIHR is committed to responding to your submission of information within two weeks or we will update you on progress.

The enclosures to this letter detail the feedback on your application, the processes to be undertaken during the next steps, and additional information relating to your award. Your contact person within NIHR is Harry Gibbs, who will be working with you on the contract. Please could you let Harry know if you are unable to respond to the Funding Committee and Finance and IP queries by the deadlines noted above.

Yours sincerely,

Lynn Kerridge
Chief Executive Officer
NIHR Evaluation, Trials and Studies Coordinating Centre

Enclosures:
- Funding Committee, Finance and IP Feedback
- Research Management and Approvals Information
