# Supplementary material for: Remote STrOke Rehabilitation (ReSTORe): study protocol for a randomised controlled trial
Source: Trials. 2026 Aug 13;27:657. doi: 10.1186/s13063-026-09952-5 (PMC13749901; doi:10.1186/s13063-026-09952-5)
Supplement: Supplementary file 2 [file 13063_2026_9952_MOESM2_ESM.pdf]

East Midlands - Derby Research Ethics Committee
2 Redman Place
London
EC20 1JQ

Please note: This is the favourable opinion of the REC only and does not allow you to start your study at NHS sites in England until you receive HRA Approval

23 December 2024

Prof Gordon McGregor
Associate Clinical Professor
University Hospitals Coventry & Warwickshire NHS Trust
UHCW
Coventry
CV2 2DX

Dear Prof McGregor

**Study title:**	**Remote STrOke Rehabilitation (ReSTORe): a UK-wide randomised controlled trial**
**REC reference:**	**24/EM/0274**
**Protocol number:**	**GM641224**
**IRAS project ID:**	**342547**

Thank you for your letter of 18th December 2024, responding to the Research Ethics Committee's (REC) request for further information on the above research and submitting revised documentation.

The further information has been considered on behalf of the Committee by the Vice-Chair, Margret Vince and Lead Reviewer Ronald Jubb.

Confirmation of ethical opinion

On behalf of the Committee, I am pleased to confirm a favourable ethical opinion for the above research on the basis described in the application form, protocol and supporting documentation as revised, subject to the conditions specified below

Good practice principles and responsibilities

The UK Policy Framework for Health and Social Care Research sets out principles of good practice in the management and conduct of health and social care research. It also outlines the responsibilities of individuals and organisations, including those related to the four elements of research transparency:

1. registering research studies
2. reporting results
3. informing participants
4. sharing study data and tissue

Conditions of the favourable opinion

Confirmation of Capacity and Capability (in England, Northern Ireland and Wales) or NHS management permission (in Scotland) should be sought from all NHS organisations involved in the study in accordance with NHS research governance arrangements. Each NHS organisation must confirm through the signing of agreements and/or other documents that it has given permission for the research to proceed (except where explicitly specified otherwise).

Guidance on applying for HRA and HCRW Approval (England and Wales)/ NHS permission for research is available in the Integrated Research Application System.

For non-NHS sites, site management permission should be obtained in accordance with the procedures of the relevant host organisation.

Sponsors are not required to notify the Committee of management permissions from host organisations

Registration of Clinical Trials

All research should be registered in a publicly accessible database and we expect all researchers, research sponsors and others to meet this fundamental best practice standard.

It is a condition of the REC favourable opinion that **all clinical trials are registered** on a public registry before the first participant is recruited and no later than six weeks after. For this purpose, ‘clinical trials’ are defined as:

- clinical trial of an investigational medicinal product
- clinical investigation or other study of a medical device
- combined trial of an investigational medicinal product and an investigational medical device
- other clinical trial to study a novel intervention or randomised clinical trial to compare interventions in clinical practice.

A 'public registry' means any registry on the WHO list of primary registries or the ICMJE list of registries provided the registry facilitates public access to information about the UK trial.

Failure to register a clinical trial is a breach of these approval conditions, unless a deferral has been agreed by the HRA (for more information on registration and requesting a deferral see: Research registration and research project identifiers).

Where a deferral is agreed we expect the sponsor to publish a minimal record on a publicly accessible registry. When the deferral period ends, the sponsor should publish the full record on the same registry, to fulfil the condition of the REC favourable opinion.

If you have not already included registration details in your IRAS application form you should notify the REC of the registration details as soon as possible.

Where the study is registered on ClinicalTrials.gov, please inform deferrals@hra.nhs.uk and the Research Ethics Committee (REC) which issued the final ethical opinion so that our records can be updated.

Publication of Your Research Summary

We will publish your research summary for the above study on the research summaries section of our website, together with your contact details, no earlier than three months from the date of this favourable opinion letter. Where a deferral is agreed, a minimum research summary will still be published in the research summaries database. At the end of the deferral period, we will publish the full research summary.

Should you wish to provide a substitute contact point, make a request to defer, or require further information, please visit: Research summaries - Health Research Authority (hra.nhs.uk)

It is the responsibility of the sponsor to ensure that all the conditions are complied with before the start of the study or its initiation at a particular site (as applicable).

After ethical review: Reporting requirements

The attached document "After ethical review – guidance for researchers" gives detailed guidance on reporting requirements for studies with a favourable opinion, including:

- Notifying substantial amendments
- Adding new sites and investigators
- Notification of serious breaches of the protocol
- Progress and safety reports
- Notifying the end of the study, including early termination of the study
- Final report
- Reporting results

The latest guidance on these topics can be found at Managing your approval - Health Research Authority (hra.nhs.uk)

Ethical review of research sites

NHS/HSC sites

The favourable opinion applies to all NHS/HSC sites taking part in the study, subject to confirmation of Capacity and Capability (in England, Northern Ireland and Wales) or management permission (in Scotland) being obtained from the NHS/HSC R&D office prior to the start of the study (see "Conditions of the favourable opinion" below).

Non-NHS/HSC sites

I am pleased to confirm that the favourable opinion applies to any non-NHS/HSC sites listed in the application, subject to site management permission being obtained prior to the start of the study at the site.

Approved documents

The final list of documents reviewed and approved by the Committee is as follows:

Document	*Version*	*Date*
Copies of materials calling attention of potential participants to the research [ReSTORe Flyer]	1.0	13 November 2024
Copies of materials calling attention of potential participants to the research [ReSTORE Email Template Be Part of Research Charity]	1.0	13 November 2024
Copies of materials calling attention of potential participants to the research [ReSTORe_Promotional Video Script]	1.0	13 November 2024
GP/consultant information sheets or letters [ReSTORe GP Letter]	1.0	13 November 2024
Interview schedules or topic guides for participants [ReSTORe Indicative topics for Process Evaluation Interviews]	1.0	13 November 2024
IRAS Application Form [IRAS_Form_20112024]		20 November 2024
Letter from funder [NIHR157510 Start Up Letter]	1.0	30 November 2023
Letter from sponsor [ReSTORe Sponsorship Letter]	2.2	01 April 2023
Letters of invitation to participant [ReSTORe Patient Invitation Letter]	1.0	13 November 2024
Other [ReSTORe Messages to participants_Database]	1.0	14 November 2024
Other [ReSTORe_Support Slides]	1.0	18 November 2024
Other [ReSTORe_Participant Workbook]	1.0	14 November 2024
Other [ReSTORe_Data Flow Map]	1.0	14 November 2024
Other [ReSTORe Data Flow Map Table]	1.0	13 November 2024
Other [Electronic Eligibility Form]	1.3	13 November 2024
Other [clinical_trials_twimc_letter_2024-25 (1)]		
Other [Response to PO]		
Participant consent form [ReSTORe Consent Form]	1.0	13 November 2024
Participant consent form [ReSTORe Process Evaluation Consent Form]	1.0	14 November 2024
Participant information sheet (PIS) [ReSTORe Patient Information Sheet]	1.0	12 December 2024
Participant information sheet (PIS) [ReSTORe Easy Read Participant Information Sheet]	1.0	12 December 2024
Participant information sheet (PIS) [ReSTORe Process Evaluation Information Sheet]	1.0	12 December 2024
Participant information sheet (PIS) [ReSTORe easy read Participant Information sheet TC]	0.4	12 December 2024
Participant information sheet (PIS) [ReSTORe Process Evaluation Information Sheet TC]	0.26	12 December 2024
Participant information sheet (PIS) [ReSTORe Patient Information Sheet TC]	0.4	12 December 2024
Research protocol or project proposal [ReSTORe Protocol]	1.0	14 November 2024

Summary CV for Chief Investigator (CI) [Gordon McGregor Short CV April 2024]	1.0	14 April 2024
Summary, synopsis or diagram (flowchart) of protocol in non technical language [ReSTORe_Study Flow Diagram]	1.0	14 November 2024
Validated questionnaire [ReSTORe Basline Questionnaire]	1.0	13 November 2024
Validated questionnaire [ReSTORe 3 month Questionnaire]	1.0	13 November 2024
Validated questionnaire [ReSTORe 6 month Questionnaire]	1.0	13 November 2024
Validated questionnaire [ReSTORe 12 month Questionnaire]	1.0	13 November 2024

Statement of compliance

The Committee is constituted in accordance with the Governance Arrangements for Research Ethics Committees and complies fully with the Standard Operating Procedures for Research Ethics Committees in the UK.

User Feedback

The Health Research Authority is continually striving to provide a high quality service to all applicants and sponsors. You are invited to give your view of the service you have received and the application procedure. If you wish to make your views known please use the feedback form available on the HRA website: Quality assurance - Health Research Authority (hra.nhs.uk)

HRA Learning

We are pleased to welcome researchers and research staff to our HRA Learning Events and online learning opportunities– see details at: Learning - Health Research Authority (hra.nhs.uk)

IRAS project ID: 342547   Please quote this number on all correspondence

With the Committee's best wishes for the success of this project.

Yours sincerely

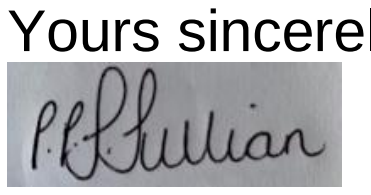

Mrs Janet Mallett
Chair

Email:derby.rec@hra.nhs.uk

Enclosures: "After ethical review – guidance for researchers"

Copy to: Mrs Lucy Eggleston

Lead England: approvals@hra.nhs.uk
